# When Emotion Blinds: A Spatiotemporal Competition Account of Emotion-Induced Blindness

**DOI:** 10.3389/fpsyg.2012.00438

**Published:** 2012-11-07

**Authors:** Lingling Wang, Briana L. Kennedy, Steven B. Most

**Affiliations:** ^1^Department of Psychology, University of DelawareNewark, DE, USA; ^2^School of Psychology, The University of New South WalesSydney, NSW, Australia

**Keywords:** emotion-induced blindness, attention, perception, spatiotemporal competition, biased competition, emotion, visual awareness

## Abstract

Emotional visual scenes are such powerful attractors of attention that they can disrupt perception of other stimuli that appear soon afterward, an effect known as *emotion-induced blindness*. What mechanisms underlie this impact of emotion on perception? Evidence suggests that emotion-induced blindness may be distinguishable from closely related phenomena such as the orienting of spatial attention to emotional stimuli or the central resource bottlenecks commonly associated with the attentional blink. Instead, we suggest that emotion-induced blindness reflects relatively early competition between targets and emotional distractors, where spontaneous prioritization of emotional stimuli leads to suppression of competing perceptual representations potentially linked to an overlapping point in time and space.

Most aspects of the environment resonate with emotional meaning, so an understanding of perception in the real world necessitates understanding how it is impacted by emotion. Evidence suggests that emotional stimuli themselves attract attention more robustly and are more readily detected than are non-emotional stimuli (Anderson and Phelps, 2001; Öhman et al., 2001; Anderson, 2005; Vuilleumier and Huang, 2009), but less well understood is the impact of emotional stimuli on the perception of neighboring non-emotional information. In a sense, this dimension of perception-emotion interactions is especially relevant to everyday life, where – whether one is a soldier on patrol, an emergency room technician, or a highly anxious individual surrounded by perceived threats – it is important to attend to and process non-emotional information despite the emotional context.

Unfortunately, the literature on perception-emotion interactions often seems to contradict itself, with some studies showing that emotional stimuli impair perception of contextually neighboring targets and other studies showing that emotional stimuli enhance perception of such targets. In the former case, for example, studies have shown that when people search for a single target embedded in a rapid, serially presented stream of pictures, the presence of a task-irrelevant emotional picture robustly impairs target perception for about a half-second, a phenomenon labeled *emotion-induced blindness* (e.g., Most et al., 2005; Most and Jungé, 2008; Most and Wang, 2011; Kennedy and Most, 2012). In contrast, other studies have shown that the presentation of a task-irrelevant emotional face can subsequently enhance contrast sensitivity (a function of early vision; Phelps et al., 2006; Bocanergra and Zeelenberg, 2009) and can facilitate visual search for targets (Becker, 2009). Findings that emotional stimuli can benefit subsequent target perception are consistent with a recently proposed “arousal-biased competition” account, which posits that emotional stimuli bias subsequent perceptual competition in favor of high-priority stimuli (which can be classified as “high-priority” by virtue of either their inherent salience or their goal-relevance; Mather and Sutherland, 2011).

But what of the cases where emotional stimuli disrupt perception? Why should emotional stimuli enhance subsequent perception of targets on some occasions but disrupt it on others? In a sense, the competition processes posited within the arousal-biased competition account might suggest insights into emotion-induced blindness, as emotional stimuli themselves could be construed as high-priority stimuli that compete with neighboring targets. Indeed, recent work on emotion-induced blindness in our lab has revealed some clues into the nature of such competition. To anticipate, our evidence suggests that emotion-induced blindness may stem from competition between targets and emotional distractors and that the phenomenon primarily arises when targets and emotional distractors jockey to be the dominant representation linked to a given point in space and time.

## Emotion-Induced Blindness

**Figure 1:**
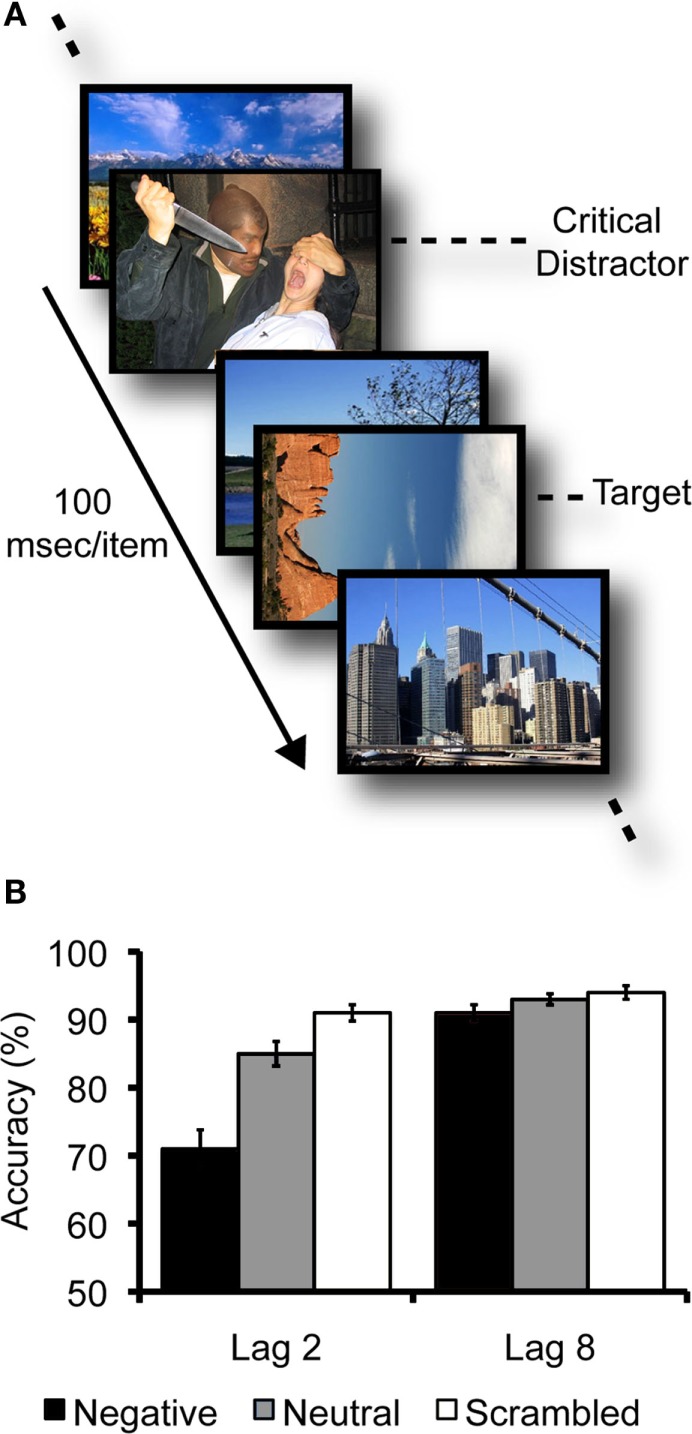
**(A)** Example of part of a typical emotion-induced blindness trial, where items are presented serially at a rate of 100 ms/item. Here, the target is a landscape picture rotated 90° clockwise. The critical distractor is an emotionally negative picture that appears two items before the target (Lag 2). **(B)** A typical pattern of data (means and standard errors) from an emotion-induced blindness experiment (from Most et al., 2005, Experiment 1). At Lag 2, accuracy in reporting the target’s orientation was worse following emotional distractors than following neutral or scrambled distractors. However, by Lag 8, performance in all distractor conditions had recovered to above 90% accuracy.

In a series of studies showing emotional disruption of conscious perception, participants viewed rapid serial visual presentations (RSVPs) of upright landscape and architecture photos at a rate of 10 images per second. They were instructed to search within each stream for a landscape or architecture photo that was rotated 90° clockwise or counterclockwise and to report its orientation (see Figure 1A). Depending on the trial, a task-irrelevant emotionally negative, neutral, or scrambled negative picture preceded the target picture by either two (lag 2) or eight (lag 8) items (Most et al., 2005). Emotionally negative distractors depicted aversive, highly arousing scenes such as threatening animals, violence, or medical trauma, whereas neutral images depicted people or animals in ways that were not emotionally evocative. Scrambled versions of the negative distractors served to control for the impact of low-level visual properties such as color and luminance. Despite the rapid presentation rate, participants were highly accurate in reporting the target’s orientation at lag 8 (when the distractor and the target appeared almost 1 s apart) regardless of distractor condition. However, at the earlier lag, emotionally negative distractors induced greater deficits in target processing than did the scrambled and neutral distractors (see Figure 1B). This pattern – emotion-induced blindness – appears to reflect a disruption of conscious perception rather than disrupted maintenance of information in visual working memory, as the size of the effect is comparable regardless of whether participants respond immediately or withhold their response for a brief delay (Kennedy and Most, 2012).

The fact that the scrambled versions of the negative pictures did not induce spontaneous target perception impairments suggests that the impairments elicited by the negative images stemmed from their emotional nature rather than their low-level visual features. This conclusion received further support from a study in which emotionally neutral pictures that participants had learned to associate with an aversive burst of white noise induced similar target perception impairments (Smith et al., 2006). Both emotionally negative and emotionally positive distractors appear capable of driving the effect as long as they elicit a response of relatively high arousal: in one set of experiments, the emotional distractors were erotic scenes – which are generally rated as emotionally positive and highly arousing by both men and women (Bradley et al., 2001; Lang et al., 2001) – and these stimuli consistently elicited emotion-induced blindness effects similar to those caused by negative distractors (Most et al., 2007).

## Differentiating Emotion-Induced Blindness from Related Phenomena

In some respects, emotion-induced blindness is surprising within the context of the extant literature. As mentioned above, the phenomenon stands in contrast to findings that emotional stimuli can facilitate perceptual processing of subsequent stimuli (Phelps et al., 2006; Becker, 2009; Bocanergra and Zeelenberg, 2009). Because all stimuli appear in the same spatial location in most emotion-induced blindness tasks, the phenomenon also appears to contrast with evidence that emotional stimuli facilitate the processing of subsequent stimuli at their location by attracting or holding spatial attention there (e.g., MacLeod et al., 1986; Mogg and Bradley, 1999; Fox et al., 2001; Jiang et al., 2006; Van Damme et al., 2008). One possibility is that emotion-induced blindness reflects mechanisms other than spatial attention or those involved in enhancing early perception. But if so, what mechanisms might be involved?

On the surface, it seems likely that emotion-induced blindness stems from the same mechanisms as the *attentional blink* (AB; e.g., Raymond et al., 1992; Chun and Potter, 1995), a failure of conscious perception that is widely studied in the visual cognition literature (and which served as the model for the emotion-induced blindness task). In a typical AB task, participants report two targets embedded in a rapid stream of non-targets (e.g., the identities of two letters embedded in a stream of digits). If the two targets appear far enough apart from each other in time, then people can generally report both targets despite the rapid presentation speed. However, if the second target appears within about half a second after the first target, then people can generally report the first but not the second target (cf. Potter et al., 2002). Dominant models of the AB differ somewhat from each other in their details, but most converge on the notion that the AB largely reflects the disruption or distraction of relatively central, late-stage perceptual mechanisms, whether such mechanisms involve a failure of consolidation into visual working memory (e.g., Chun and Potter, 1995), a failure of retrieval from memory (e.g., Shapiro et al., 1994), or disruption of an attentional filter responsible for distinguishing targets from non-targets (e.g., Di Lollo et al., 2005), among other accounts (but also see Giesbrecht et al., 2007, for evidence of the flexibility of the AB locus).

Based on such accounts of the AB, it might be expected that emotion-induced blindness reflects the disruption of some relatively central, high-level mechanism. However, in a recent series of experiments, a surprising pattern of results suggested that – despite its surface-level similarity to the AB – emotion-induced blindness might stem from mechanisms other than those often presumed to drive the AB. In these experiments, participants searched for a target that could appear in one of two simultaneously presented, vertically aligned rapid streams instead of within a single stream. The target was equally likely to appear in either stream, and the critical distractor (a neutral or emotionally negative picture) was equally likely to appear in the same stream as the target or in the opposite stream. Results revealed that target perception was worse following an emotional distractor only when the critical distractor appeared in the same stream as the target (Most and Wang, 2011). If emotion-induced blindness stemmed from the disruption or distraction of a relatively late-stage, central processing bottleneck, then the perceptual disruption should have been equivalent regardless of the spatial relationship between the targets and critical distractors, as seems to be the case with the AB (e.g., Shih, 2000; Lunau and Olivers, 2010; but see Kristjánsson and Nakayama, 2002). The fact that the emotion-induced impairment of target perception was greater at, compared to away from, the location of the emotional distractor suggests that the mechanisms underlying emotion-induced blindness may be dissociable both from central bottlenecks involved in the AB and from the spatial attention mechanisms that have been the focus of most emotion-perception research over the past several decades.

Notably, further evidence for a dissociation between spatial attention mechanisms and those underlying emotion-induced blindness emerged from an emotion-induced blindness experiment in which conditions that were more or less conducive to spatiotemporal competition between targets and emotional distractors were manipulated. In most emotion-induced blindness tasks, the targets and critical distractors are both embedded in the middle of a rapid stream, rendering their temporal order hard to judge and increasing the likelihood of the perceptual system linking them to a common point in time and space. However, in this follow-up experiment, the targets were sometimes the last item in their stream (Most and Wang, 2011, Experiment 2). With no subsequently appearing items masking the targets, the targets could persist in iconic memory and their temporal relationship with the critical distractors was rendered unambiguous. Under these conditions, the spatial pattern of emotion-induced impairment reversed, with target accuracy now worse away from – rather than at – the location of the emotional distractor. This reversed pattern is consistent with the large corpus of studies suggesting that emotional distractors capture spatial attention and delay or otherwise impair processing of targets at other locations. In other words, when the hypothesized impact of spatiotemporal competition was minimized, a dissociable impact of spatial attention appeared to emerge.

## A Spatiotemporal Competition Account of Emotion-Induced Blindness

Although the localization of emotion-induced blindness to the location of an emotional distractor appears to run counter to what might have been predicted on the basis of the spatial attention literature, it accords well with research on *localized attentional interference* (Mounts, 2000, 2005; Mounts and Gavett, 2004; Mounts and Tomaselli, 2005; McCarley and Mounts, 2007; McCarley et al., 2007; Mounts et al., 2007). This refers to the finding that processing of one stimulus can impair processing of a second stimulus that appears in close spatial proximity and that this impairment grows stronger with decreasing distance between the two targets (also see Cave and Zimmerman, 1997; Caputo and Guerra, 1998; Bahcall and Kowler, 1999; Kristjánsson and Nakayama, 2002; Theeuwes et al., 2004; Doran and Hoffman, 2010). Given the degree to which such localized interference seems similar to the spatial pattern of emotion-induced blindness, the mechanisms underlying it may suggest insights into the nature of emotion-induced blindness.

Patterns of localized attentional interference are consistent with an overarching “biased competition” model of attentional selection (e.g., Desimone and Duncan, 1995; Desimone, 1998), one of the foundations of which is the recognition that visual stimuli in a cluttered visual environment compete with each other to drive the responses of neurons in the visual system. According to this account, two or more simultaneously appearing stimuli will evoke neural patterns of activation in parallel. When the stimuli lie far apart enough in the visual field, they may evoke activity in minimally overlapping neuron populations. However, the smaller the distance between the stimuli, the greater the overlap in the neuron populations activated, leading to increased competition between the neural representations. In this situation, selective attention is conceptualized as a biasing of the competition in favor of one stimulus over the others. This competition can be biased in bottom up fashion – in favor of items that are visually salient – or by top-down strategy – in favor of items that are goal-relevant (Desimone and Duncan, 1995; Desimone, 1998). Recordings of neural activity have provided evidence consistent with the biased competition account. For example, visual cortical neurons that are highly responsive to one stimulus are less responsive when a second, competing stimulus simultaneously occupies their receptive fields, but attention to either of the stimuli leads to a neural response similar to that observed when the attended item appears alone (e.g., Chelazzi et al., 2001). Notably, receptive fields are small in early regions of the visual cortex, where neural activity appears to be driven largely by low-level visual properties; however, they grow larger in later, more anterior visual regions, which have been found to be more globally responsive to complex stimuli such as objects and faces (Desimone and Gross, 1979; Gattass et al., 1981, 1988; Kastner and Ungerleider, 2000; Kastner et al., 2001). Competition between neural representations has been observed in a number of regions, including V1, V2, V4, and inferotemporal cortex, the hierarchical organization of which suggests that competitive spatiotemporal interactions could occur not only at the level of discrete features, but also at the level of meaningful representations. Since the time that the biased competition model of attention was first proposed, empirical and theoretical advances have extended and refined it, as reflected in (for example) more recent “normalization” and “feature-similarity gain” models (e.g., Treue and Martinez-Trujillo, 1999; Martinez-Trujillo and Treue, 2004; Lee and Maunsell, 2009; Reynolds and Heeger, 2009). Nevertheless, the biased competition account provides a useful framework for understanding the relatively limited number of emotion-induced blindness findings to date, with a fuller consideration of the distinctions between related models and their implications for emotion-induced blindness likely to provide ever more insight as research on this topic progresses.

In an insightful review, Keysers and Perrett (2002) noted that – when presented rapidly enough – temporally neighboring stimuli within RSVP streams are likely to give rise to spatiotemporal competition despite their sequentially presented nature. This is because, even though the stimuli do not appear simultaneously with each other, they elicit neural responses that themselves overlap in time. Framed within this context, spatial localization of emotion-induced blindness makes sense. When a target appears soon after an emotional distractor (or soon before; Most and Jungé, 2008), the stimuli compete to be the dominant representation linked to an overlapping point in time and space. Because of the human tendency to spontaneously prioritize emotional stimuli, the distractor frequently dominates and suppresses visual processing of the target.

Notably, if emotion-induced blindness arises due to competition between targets and emotional distractors, then it may be possible to apply manipulations to either strengthen the bias for emotional distractors or boost the competitive edge of targets, thereby modulating – possibly via reentrant mechanisms (e.g., Lamme and Roelfsema, 2000) – the degree of emotion-induced blindness observed. In fact, this appears to be the case. For example, in one experiment participants were informed in some blocks that their target could be a rotated picture of either (a) a building or (b) a landscape with no building, and in the remaining blocks they were informed that their rotated target would always be a picture of a building (Most et al., 2005, Experiment 2). The latter case – labeled the “specific attentional set” condition – enabled participants to establish a more concrete attentional template of what their target would look like, and the results revealed that emotion-induced blindness decreased in this condition, at least among participants who had scored low in a measure associated with trait anxiety. This is consistent with the notion that attentional competition can be biased via goal-relevant information held in working memory (e.g., Desimone and Duncan, 1995; Desimone, 1998). This instruction did not reduce emotion-induced blindness among participants who had scored high in the anxiety-related measure, however, perhaps because for them the bias to prioritize emotional stimuli was more difficult to overcome.

Indeed, in another set of experiments, participants’ level of anxiety was directly manipulated, with participants who reported high levels of unease exhibiting greater emotion-induced blindness than those who did not. In this set of experiments, male and female romantic partners were seated at computers a few feet away from each other. The female partner engaged in an emotion-induced blindness task, first while her male partner rated the attractiveness of landscape pictures and then while he rated the attractiveness of women who ostensibly were single and on campus (although, in truth, the pictures had been gathered from the internet and had no known relationship with the university). At the end of the experiment, the female participants were asked to rate their level of unease about the fact that their partner had been rating other women; in two separate experiments, there was a robust correlation between self-rated unease and emotion-induced blindness (Most et al., 2010). Intriguingly, this correlation emerged only when the distractors were emotionally negative, not when they were emotionally positive. Moreover, self-rated unease predicted emotion-induced blindness only during the time that the male partner was rating the attractiveness of other women and not when he was rating the attractiveness of landscapes, helping to rule out individual differences unrelated to the manipulation (e.g., the possibility that participants who experienced unease also happened to be more sensitive to emotionally negative images in general). In short, such evidence is consistent with the notion that emotion-induced blindness is driven by competition between target and emotional distractor representations: whereas the competition can be skewed in favor of targets by providing more descriptive information about their visual appearance (Most et al., 2005, Experiment 2), it appears that anxiety can enhance the bias in favor of emotional distractors (Most et al., 2010).

## Attentional Capture vs. Emotional Capture

An important question regarding the nature of emotion-induced blindness is whether the mechanisms underlying it are simply identical to those that would be triggered by any attention-capturing stimulus, or whether emotion-induced blindness instead stems from processes triggered uniquely by the heightened meaningfulness of the emotional distractor. While attention can be captured by emotional stimuli, it can also be captured by stimuli that either share a defining feature with the target (i.e., that match participants’ “attentional set;” Folk et al., 1992; Folk and Remington, 1998) or by stimuli that are featurally salient or unique in the environment (e.g., Yantis and Jonides, 1990; Theeuwes, 1991, 1992, 1994; Yantis, 1993). Within RSVP tasks, such non-emotional, attention-grabbing stimuli have been found to induce spontaneous attentional blinks for subsequent targets (Spalek et al., 2006; Folk et al., 2007). Given the ability of non-emotional, attention-capturing stimuli to induce spontaneous perceptual disruptions resembling those caused by emotional stimuli, it may be that emotion-induced blindness simply reflects attentional capture rather than a more elaborate process through which emotion impacts perception.

In a recent series of studies, we capitalized on the spatially localized nature of emotion-induced blindness to examine whether target perception impairments caused by emotional and non-emotional, but attention-grabbing, distractors share common underlying mechanisms (Wang and Most, 2011; Wang and Most, in preparation). If emotion-induced blindness stems simply from the tendency of attention to spontaneously orient to emotional stimuli, then target perception deficits caused by the non-emotional, attention-grabbing distractors should also be spatially localized. To this end, we varied the nature of critical distractors in the dual-stream RSVP paradigm. In a set of two experiments, participants searched for a red letter embedded within one of two simultaneously presented rapid streams of white letters, and the critical distractor (which could appear in either stream) was either a red digit or a green letter. In a third experiment, participants searched for a rotated color landscape photo embedded in one of two simultaneous streams of grayscale landscape photos, and the critical distractor was an upright color landscape photo (thereby matching participants’ attentional set for color). In all three cases, the non-emotional, but attention-grabbing, distractors impaired subsequent target perception, but this impairment was not spatially localized.

In a fourth experiment designed to ensure identical task demands across conditions, the target was a rotated color landscape photograph embedded among rapidly presented, upright grayscale landscape photos, and the distractor was either an upright color landscape photo, an emotional color picture, or a neutral non-landscape color photo. Target perception impairments caused by the landscape and neutral color photos were not spatially localized, but the impairments caused by the emotional pictures were specific to the distractors’ location (Wang and Most, 2011; Wang and Most, in preparation).

In sum, although non-emotional, attention-grabbing distractors disrupted target perception, the spatially localized nature of the impairment seemed to emerge specifically in the temporal wake of emotional distractors. Our lab is currently in the process of further verifying these results and testing whether they can be accounted for by mechanisms other than spatiotemporal competition. Thus far, the data are consistent with the suggestion that emotion-induced blindness does not stem simply from the tendency of attention to orient to emotional distractors. Neuroimaging studies along these lines would likely be fruitful, as the behavioral findings to date yield intriguing predictions. Framed in terms of neural architecture, competition between targets and emotional distractors may involve relatively anterior visual brain regions that are responsive to complex, meaningful representations, and such regions may function as the neural locus where emotional stimuli gain a competitive edge.

## Conclusion

Although emotional stimuli can sometimes facilitate perception of subsequent items, they can also disrupt perception, yielding results that seem contradictory at first glance. Research on emotion-induced blindness and its underlying mechanisms can help reconcile such discrepancies. For example, evidence suggests that, consistent with a biased competition account of attention, emotional disruption of perception may occur primarily when emotional distractors and targets appear in such way as to be linked by the visual system to overlapping points in time and space. In the absence of such spatiotemporal competition, emotional stimuli have sometimes been found to enhance perception (e.g., see Bocanergra and Zeelenberg, 2009; Ciesielski et al., 2010). Follow-up neurophysiological studies will greatly improve our understanding of the neural locus of this competition; behavioral evidence so far suggests that it may functionally lie earlier than consolidation into working memory (evidenced, for example, by patterns of spatial localization) but late enough in perceptual processing to involve competition between meaningful representations.

Of course, the impact of emotion on perception is multi-faceted. Depending on the intensity of the emotional stimuli, the conditions under which they appear, or the personality of the perceiver, there may be circumstances where emotional stimuli impair (or facilitate) perception of neighboring targets through relatively central, late-stage mechanisms as well (e.g., consolidation into visual working memory). The evidence reviewed in the present discussion highlights potential spatiotemporal competition mechanisms; further characterization of the loci at which emotion can impact visual processing holds promise for more fully understanding the myriad ways that it can shape our conscious perception of the world.

## Conflict of Interest Statement

The authors declare that the research was conducted in the absence of any commercial or financial relationships that could be construed as a potential conflict of interest.
